# Economic Evaluation and Catheter-related Bloodstream Infections

**DOI:** 10.3201/eid1306.070048

**Published:** 2007-06

**Authors:** Kate Halton, Nicholas Graves

**Affiliations:** * The Centre for Healthcare Related Infection Surveillance and Prevention, Brisbane, Queensland, Australia; † Queensland University of Technology, Brisbane, Queensland, Australia

**Keywords:** Catheterization, central venous catheters, intensive care, costs and cost analysis, decision making, decision support, techniques, models, economic, perspective

## Abstract

Catheter-related bloodstream infections are a serious problem. Many interventions reduce risk, and some have been evaluated in cost-effectiveness studies. We review the usefulness and quality of these economic studies. Evidence is incomplete, and data required to inform a coherent policy are missing. The cost-effectiveness studies are characterized by a lack of transparency, short time-horizons, and narrow economic perspectives. Data quality is low for some important model parameters. Authors of future economic evaluations should aim to model the complete policy and not just single interventions. They should be rigorous in developing the structure of the economic model, include all relevant economic outcomes, use a systematic approach for selecting data sources for model parameters, and propagate the effect of uncertainty in model parameters on conclusions. This will inform future data collection and improve our understanding of the economics of preventing these infections.

## Abstract 2

A comprehensive understanding of the economics of preventing catheter-related bloodstream infections is needed.

Catheter-related bloodstream infections (CR-BSI) occur at an average rate of 5 per 1,000 catheter days in intensive-care units in the United States (*1*), resulting in 80,000 episodes of CR-BSI per year (*2*). This situation leads to increased patient illness, length of stay, and costs of care (*3*,*4*) and possibly additional deaths (*5*). Empiric evidence (*6*) suggests that >50% of these infections could be prevented. The evidence for the effectiveness of numerous single and multimodule interventions has been reviewed (*2*,*7*), leaving the decision maker with the complex task of selecting the best infection-control programs. This decision should be informed by data on the effectiveness of an intervention as well as an understanding of the cost implications (*8*).

An effective strategy that reduces the risk for CR-BSI will generate health benefits from avoided illness and possibly reduced deaths. At the same time, preventing infections will save costs, and these are offset against cost increases from implementing the strategy. The aggregate of these costs will be either positive (cost-increasing) or negative (cost-saving). An effective program that saves costs must be implemented so as not to waste resources and harm patients at the same time. An effective program that increases costs should be subject to a cost-effectiveness test (e.g., <$50,000 per life year gained) and, if successful, it should be given serious consideration by policymakers. This information can be found in full economic evaluations in which changes to costs and health benefits for a novel strategy are compared with a relevant comparator such as current practice (*8*,*9*). This enables us to identify the course of action that offers optimal returns from our investment of resources.

With the current spending on healthcare in the United States being >15% of the gross domestic product (*10*), the US Food and Drug Administration, as well as the regulatory agencies for the United Kingdom, Australia, and Canada, now require additional programs or therapies to demonstrate cost-effectiveness. The message is clear: new healthcare investments should promote efficiency in resource allocation, not detract from it.

The existing economics literature for CR-BSI includes 2 approaches to full economic evaluation. First are trial-based evaluations in which values for parameters such as costs and health benefits are derived from a single data-collection exercise. Second are modeling studies for which values for these parameters are obtained from a variety of sources and combined in a decision-analytic model. The advantages and disadvantages of each have been discussed (*11*). A major advantage of model-based evaluations is the ability to include long-term cost and death outcomes not observed within the period of a clinical trial. Also, interventions that have not been or cannot be directly compared in a clinical trial can be evaluated side by side in modeling studies. These evaluations allow consideration of all relevant competing infection control interventions and not just a single novel strategy compared with existing practice. Finally, model-based evaluations are more generalizable and can be used to evaluate the cost-effectiveness of an intervention in a real-life context not represented by the results of a trial. For these reasons they are the increasingly the preferred approach to the economic evaluation of healthcare interventions (*12*). However, care is needed and only high-quality, appropriately designed and unbiased models should be published and used for policymaking (*11*).

The aims of our study are to summarize the existing literature on model-based economic evaluation of interventions to prevent CR-BSI and then critique this literature, focusing on 2 questions. 1) How useful are the evaluations in terms of how the research questions and findings align with the information needed to make good decisions? 2) What is the quality of the evaluations, in particular, whether the quality of the model structure, the source of parameter data and its incorporation into the model, and the techniques used to evaluate the model are such that the evidence provided is convincing to decision makers? Ultimately, we aim to judge the value of this body of literature in helping us understand the economics of preventing CR-BSI and identify priorities for future research that will lead to a deeper understanding of this topic.

## Methods

We reviewed data published between 1990 and November 2005. Searches were conducted in Medline, the Cumulative Index to Nursing and Allied Health Literature, Biologic Abstracts, Academic Search Elite, and Econlit by using the medical subject headings catheterization central venous, costs and cost analysis, and infection; or text keywords catheter and central, cross-referenced with infection, bacteremia, or sepsis, and cost-effective, cost-benefit, or cost-utility. We searched the Centre for Reviews and Dissemination databases (www.york.ac.uk/inst/crd) by using the same subject keywords and limiting the search to economic evaluations. In addition, the reference lists of retrieved articles and review articles in this field of research (*13*–*16*) were searched to identify published articles that met predefined inclusion and exclusion criteria (Table 1).

**Table 1:** Inclusion and exclusion criteria for review

Inclusion criteria
Had a full publication or manuscript for review
Conducted a full economic evaluation which valued both costs and benefits of the intervention
Based on a decision-analytic model
Evaluated at least 1 infection-control intervention aimed at reducing incidence of catheter-related bloodstream infection relative to a baseline scenario
Evaluated the intervention with respect to short-term (<21 d), nontunneled, central venous catheters
Based in an adult patient population
Written in English
Exclusion criteria
Cost-analysis studies only
Did not use a comparator
Based on a clinical trial (e.g., randomized controlled trial or pre-post intervention study) or a case study
Did not contain an original analysis (e.g., editorials, reviews)
Contained purely hypothetical data (e.g., methods articles)
Did not provide full details on methods (e.g., letters)
Based in a pediatric patient population
Evaluated interventions aimed at long-term or tunneled or peripherally placed central venous catheters
Evaluated therapeutic or diagnostic interventions, as opposed to preventive interventions

To assess the usefulness of the economic evaluations included, summary data for each were extracted by using an audit tool based on the Harvard Cost-Effectiveness Analysis Registry data abstraction forms (*17*). The data extracted included a description of the intervention(s) and population studied, the research question, the structure of the economic model and assumptions used, the data used to inform model parameters, the outcomes considered, and the results and conclusions, including the results of sensitivity analyses. All US dollar figures were adjusted to 2005 prices by using the Bureau of Labor Statistics Consumer Price Index specific to Medical Care (www.bls.gov/cpi), although any common year could have been assumed. When the cost year used for the analysis was not stated, it was assumed to be 1 year before publication. This assumption will not affect evaluation of the analysis.

To assess the quality of the economic evaluations, we used a set of good practice criteria for decision analytic modeling (*18*). Four criteria are used to assess the structure of the model; 6 criteria to assess how data were sourced and incorporated, including approaches to sensitivity analysis; and 1 criterion to judge how the model was evaluated in terms of its own consistency. These 11 criteria were applied as a series of questions that focused on the relevance and coherence of the modeling approach taken in each evaluation, rather than as a prescriptive checklist.

The quality of the data used to inform model parameters was also assessed by using the modified version (*19*) of the potential hierarchies of data sources for economic analyses (*20*). Each component of the decision model was assessed: clinical effect size, baseline clinical data, adverse events, resource use, costs, and utilities. The quality of data sources is ranked from 1 to 6 with the highest quality of evidence ranked 1. Rankings for evidence pertaining to clinical effect size are comparable with the concept of levels of evidence as used in evidence-based medicine (*21*) and Cochrane reviews (*22*). For each article, the highest level of evidence used for each parameter was recorded.

## Results

A total of 106 abstracts were identified, and 8 met the inclusion criteria (*23*–*30*). The reasons for exclusion are shown in the Figure.

**Figure:**
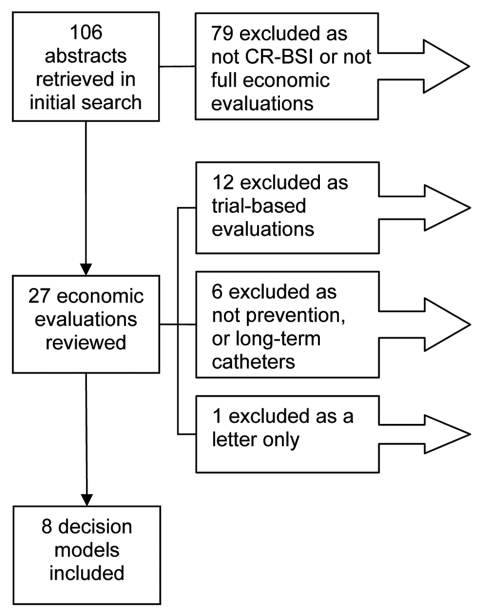
Reports included in the review. CR-BSI, catheter-related bloodstream infections. The 19 economic evaluations excluded from the review are shown in the Appendix.

### Usefulness of Evaluations

Six interventions were evaluated (Table 2); antimicrobial drug–coated catheters were included in 3 separate analyses (*27*,*29*,*30*). One intervention was compared with current practice for all studies, except those of Shorr et al. (*29*) and Ritchey et al. (*28*), who evaluated 3 types of antimicrobial drug–coated catheter and 3 different catheter replacement regimens, respectively. No direct comparisons were made across intervention types, e.g., use of an antiseptic catheter versus introduction of chlorhexidine as a skin preparation, and no evaluations assessed multiple concurrent interventions or bundles. The authors of 6 evaluations (*23*,*24*,*26*,*27*,*29*,*30*) found the intervention to be effective in preventing CR-BSI and cost-saving (Table 3), and the authors of 2 other evaluations (*25*,*28*) generated data to calculate incremental cost-effectiveness ratios.

**Table 2:** Summary of economic evaluations of interventions to prevent CR-BSI included in the review*

Intervention	Comparator	Analysis	Perspective	Sensitivity analysis	Time horizon	Hospitalized patients	Ref.
Antimicrobial catheters							
MR CVC	CHG-SSD CVC	CUA	HC payer	PROB, OW, SC	Patient lifetime	Adults at high risk for CR-BSI likely to require a triple-lumen, noncuffed CVC for >3 d	*27*
MR CVC and CHG-SSD CVC	Standard CVC	CEA	HC payer	OW, SC, TH	Duration hospitalized	Critically ill patients requiring a CVC expected to be placed >48 h	*29*
CHG-SSD CVC	Standard CVC	CEA	HC payer	PROB, OW, SC, TH	Duration hospitalized	Patients at high risk for catheter-related infections requiring short-term use (2–10 d) of multilumen CVCs	30
Aseptic technique							
MSB at CVC insertion	Less stringent asepsis	CEA	Hospital	OW, SC	Duration hospitalized	Patients requiring short-term multilumen CVC (specifically, those in ICU, with immunosuppression, or receiving TPN)	*26*
Skin preparation and dressing							
CHG skin prep	PI skin preparation	CEA	Hospital	PROB, OW, SC	Duration hospitalized	Patients requiring either a PVC or CVC (considered separately) for short-term use (<10 d)	*23*
CHG dressing	Standard dressing	CEA†	Hospital	OW, MW, SC	Duration hospitalized	Patients at high risk for catheter-related infections requiring short-term use (2–10 d) of multilumen CVCs	*24*
Total parenteral nutrition							
TPN commercial bags	TPN glass bottles	CMA/CEA	Hospital	MW, TH	Duration hospitalized	Patients receiving TPN through catheter for severe bowel dysfunction secondary to Crohn disease, medical ICU patients, and surgical ICU patients	*25*
Replacement regimen							
Optimal CVC change regimen (10 d, 5 d)	3-d change regimen	CEA	Hospital	OW, MW, TH	Duration catheterized	65-year-old man in ICU with reversible disease process	*28*

*Except for the study in reference 25, which used a regression model, all studies used a decision tree. CR-BSI, catheter-related bloodstream infections; Ref., reference; MR, minocycline and rifampicin; CVC, central venous catheter; CHG-SSD, chlorhexidine gluconate/silver sulfadiazine; CUA, cost-utility analysis; HC, healthcare; PROB, probabilistic sensitivity analysis; OW, one way; SC, scenario; CEA, cost-effectiveness analysis; TH, threshold; MSB, maximal sterile barriers; ICU, intensive-care unit; TPN, total parenteral nutrition; PI, povidone-iodine; CMA, cost-minimization analysis; MW, multi way. †Crawford et al. (*24*) identified their evaluation as a cost-benefit analysis (CBA) but they conducted a cost-effectiveness analysis with health outcomes multiplied by a dollar value to produce a monetary valuation of health benefits.

**Table 3:** Results of economic evaluations of interventions to prevent CR-BSI*

Intervention	Estimated absolute incremental benefits		Estimated incremental cost	Cost/benefit ratio	Sensitivity analysis	Ref.
	Incidence CR-BSI, %	Mortality incidence, %				
Baseline: CHG-SSD catheter	Variable	Not stated	Not stated			*27*
MR catheter†	−0.7	0.009 QALYs (–0.009, 0.016)	–$83 ($109, –$205)	Cost saving	Robust	
Baseline: standard catheter	3.30	–	$469			*29*
CHG-SSD catheter	−1.94	–	–$222	Cost saving	Robust	
MR catheter	−2.79	–	–$314	Cost saving	Robust	
Baseline: standard catheter	5.20	0.78	$710			*30*
CHG-SSD catheter	−2.20 (−1.2, −3.4)	−0.33 (−0.09, −0.78)	–$262 (–$91, –$522)	Cost saving	Robust	
Baseline: less stringent asepsis	5.30	0.80	$676			*26*
Maximal sterile barriers	−2.49	−0.38	–$274	Cost saving	Robust	
Baseline: Povidone-iodine skin preparation	3.1	0.46	$265			*23*
Chlorhexidine gluconate	−1.6 (−0.6, −2.5)	−0.23 (−0.07, −0.47)	–$134 (–$21, –$286)	Cost saving	Robust	
Baseline: standard dressing	5.00	0.05	$514			*24*‡
Chlorhexidine dressing§	−2.63	−0.03	–$259	Cost saving	Robust	
Baseline: glass TPN bottles	10.0	0.50	Not stated			*25*‡
TPN bags¶	−6.67	−0.33	Not stated	$28,326/life saved	Variable	
Baseline: 5 d	–	0.92	$1,398	Not clear from source what reported cost-effectiveness ratios represented		*28*‡
3 d	–	−0.02	$8		Variable	
10 d	–	−0.13	$63		Variable	

*All estimates have been adjusted to 2005 US dollars. Values in parentheses are 95% confidence intervals. CR-BSI, catheter-related bloodstream infections; mortality, CR-BSI attributable mortality; CHG-SSD, chlorhexidine gluconate/silver sulfadiazine; QALYs, quality-adjusted life year; MR, minocycline and rifampicin; TPN, total parenteral nutrition. †Refers to results for an 8-d duration of catheterization; intervention was cost-saving for durations >8 d and could not be evaluated for <8 d. ‡Cost year for original analysis not stated; therefore, assumed 1 year before publication. §Refers to results using baseline conservative assumptions of 5% CR-BSI incidence rate, 1% CR-BSI attributable mortality rate, and $8,000 incremental CR-BSI treatment cost. ¶Refers to results using baseline conservative assumptions of 10% CR-BSI incidence rate, 5% CR-BSI attributable mortality rate, and relative reduction in risk for CR-BSI of 0.33.

Sensitivity analysis was performed in addition to baseline analysis in 5 evaluations (*23*,*26*,*27*,*29*,*30*). This provided decision makers with information on the robustness of baseline results to different parameter estimates or characterized the effect of uncertainty in model parameters on the results (*23*,*27*,*30*). In 3 cases (*24*,*25*,*28*), sensitivity analysis formed the main body of the evaluation, and decision makers faced multiple sets of results arising from different parameter estimates.

### Quality of Economic Evaluations

The extent to which the quality criteria were met for the studies varied from 1/8 for checks on the internal consistency to 8/8 for description of strategies/comparators. This assessment is shown in Table 4.

**Table 4:** Assessment of published evaluations and good practice criteria for decision models

Attributes of good practice criteria	No. models meeting criterion, n = 8
Structure	
Perspective specified	8
Description of strategies/comparators	8
Diagram of model/disease pathways	6
Development of model structure and assumptions discussed	4
Data	
Table of model input parameters presented	5
Source of parameters clearly stated	8
Model parameters expressed as distributions	3
Model assumptions discussed	7
Sensitivity analysis performed	8
Key drivers/influential parameters identified	6
Consistency	
Statement about test of internal consistency undertaken	1

### Model Structure

All authors provided a clear description of the intervention and specified the economic perspective used, which in all cases was that of the hospital or healthcare payer rather than a societal perspective. Only Shorr et al. (*29*) justified their choice of perspective. In 7 evaluations (*23*,*24*,*26*–*30*), a decision tree was used, with a diagram provided in all but 1 report (*26*). In another evaluation (*25*), a regression model was used, and only the formula used for the baseline analysis, not the extension used for sensitivity analysis, was provided. Authors of only 4 evaluations discussed the evidence or expert opinion used to develop the structure of the model (*23*,*27*,*29*,*30*).

Each evaluation used a different representation of the disease pathway in terms of the timing and nature of the relevant clinical events. For example, 1 evaluation modeled colonization as an event preceding CR-BSI (*23*), 4 considered these as mutually exclusive events (*24*,*26*,*27*,*30*), and 3 did not consider colonization (*25*,*28*,*29*). Two models included adverse events specific to the intervention (*28*,*30*), but this was not consistent across studies, with only 1 of the 3 evaluations of antiseptic-impregnated catheters including incidence of hypersensitivity reactions to the catheter (*30*). In 7 evaluations (*23*–*26*,*28*–*30*), only the outcomes that would arise during the period of hospitalization were included. In another evaluation (*27*), the time horizon described the patient’s lifetime.

### Source and Incorporation of Data

Authors of all evaluations stated the baseline data used in the model along with its source; 5 had information in a table format (*23*,*24*,*27*,*29*,*30*). Most parameter estimates came from the published literature, although 5 evaluations performed their own cost calculations for the intervention (*23*–*26*,*29*) and 1 used original patient trial data for the estimates of daily incidence and relative risk for infectious events (*27*). Seven evaluations (*23*,*25*–*30*) discussed simplifying assumptions and issues of generalizability.

For 6 evaluations (*23*,*26*–*30*), the most important model parameters were identified (Table 5), with the following 3 parameters consistently important: reduction in risk for CR-BSI caused by the intervention, baseline incidence of CR-BSI, and cost of treating a CR-BSI. The ranks of evidence used for these and other model parameters are shown in Table 6. The level of evidence used for the effectiveness of the intervention was generally high, and authors of all evaluations provided information on how they selected the data used for this parameter. However, the level of evidence used for the cost and baseline incidence of CR-BSI was generally of lower quality; little detail was given in the reports of the evaluations as to why 1 particular estimate for a parameter was chosen over another. In particular, in all evaluations, reference was made in the introduction or discussion section to relevant information on the cost and deaths attributable to CR-BSI that was not used in the analysis. This explains the wide variation in the source and value of the estimates used for parameters between the evaluations (Table 5).

**Table 5:** Variation between economic evaluations in baseline parameter estimates*

Baseline parameters	No. times identified as key parameter	No. different estimates	Minimum estimate	Maximum estimate	Median estimate
Epidemiologic					
Incidence of CR-BSI	6/8	8/8	3.1%	8.0%	5.3%
Effectiveness of the intervention	6/8	Will vary according to intervention			
Attributable mortality	2/7	5/7	5%	15%	14%
Incidence of localized insertion site infection	0/5	4/5	5%	50%	20%
Cost					
Cost of CR-BSI	6/8	6/8	US $2,820	US $13,000	US $10,531
Cost of localized insertion site infection	0/5	3/5	US $195	US $435	US $280
Cost of intervention	2/8	Will vary according to intervention			
Cost of other complications	1/3	Will vary according to complications considered			

*All cost estimates adjusted to 2005 US dollars. Values for parameters are the baseline estimate used in the model (the same patterns of variation were observed with the ranges used for sensitivity analysis). CR-BSI, catheter-related bloodstream infections.

**Table 6:** Ranks of evidence for parameters used in the decision models*

Evidence ranking	Clinical effectiveness of intervention, n = 8	Baseline incidence CR-BSI, n = 8	Attributable mortality, n = 7	Incidence localized insertion site infection, n = 5	Cost of CR-BSI, n = 8	Cost of intervention, n n = 8
High quality						
Rank 1	5	1	–	–	2	–
Rank 2	1	1	1	–	1	7
Medium quality						
Rank 3	–	1	1	–	2	–
Low quality						
Rank 4	1	4	4	4	2	–
Rank 5	–	1	1	1	–	–
Rank 6	–	–	–	–	–	–
Unclear	1	–	–	–	1	1

*CR-BSI, catheter-related bloodstream infections.

Model parameters were expressed as probability distributions for only 3 studies (*23*,*27*,*30*), even though this method provided an opportunity to appropriately describe parameter uncertainty. All 3 studies specified the choice of distribution for model parameters and the rationale for this choice. The remaining studies (*24*–*26*,*28*,*29*) used point estimates and a range for each parameter across which the estimate was varied in sensitivity analyses. Similar to the baseline estimates, no information was given on how ranges used for sensitivity analysis were decided upon, aside from a double-it and half-it approach.

### Model Evaluation

All evaluations used deterministic sensitivity analyses by varying parameters across a range of point estimates either 1 at a time (1-way) or concurrently (multiway). Four studies (*25*,*28*–*30*) reported results of threshold analyses, i.e., the value of each parameter at which the conclusions from the analysis would change, and 6 studies (*23*,*24*,*26*,*27*,*29*,*30*) reported results of scenario analyses, i.e., results where all parameters are set to favor each specific intervention in turn (Table 2). The 3 evaluations that characterized parameters as distributions (*23*,*27*,*30*) also used probabilistic sensitivity analysis, which enabled calculation of confidence intervals around their point estimates of incremental costs and benefits.

In the 6 evaluations where the intervention was cost-saving (*23*,*24*,*26*,*27*,*29*,*30*), the conclusions were robust to the sensitivity analyses. In the 2 evaluations where an incremental cost-effectiveness ratio could be calculated (*25*,*28*), different conclusions were drawn in different scenarios (Table 3). Scenario analyses used in 6 evaluations (*23*,*24*,*26*,*27*,*29*,*30*) indicated internal consistency in the models, i.e., they behaved logically and as expected. However, only 1 evaluation (*27*) made an explicit statement on internal consistency about checks performed during the model construction and analysis. Authors of 7 evaluations discussed caveats to their work (*23*–*27*,*29*,*30*).

## Discussion

We reviewed existing model-based economic evaluations of interventions to prevent CR-BSI. Given the growing use of economic evidence to inform infection control policy (*13*), the amount of this literature is likely to increase. However, critics have questioned the validity of these evaluations. McConnell et al. (*31*) suggest that “in the absence of evidence-based medicine on the effectiveness of antimicrobial central venous catheters, on the basis of clinically relevant end points, cost-effectiveness studies are an exercise in futility” We would argue that even in this situation the best possible decision still needs to be made (*11*) and that evaluations should be judged not on their ability to predict the precise value of an intervention but on the “ability of a decision model to recommend optimal decisions” (*32*). A decision not to invest in some risk-reducing intervention or program is a decision that leads to economic and clinical outcomes that are either optimal or not optimal. Economic evaluation provides a rational way for the decision maker to rank these outcomes, which in the absence of perfect information, is of more use than producing a single, potentially misleading, dollar estimate. We critiqued the existing evaluations in terms of their usefulness in providing information relevant to clinical practice. We also assessed the quality of the evaluations and explored the implication that this would have on the information provided to decision makers.

Four interventions were found to be clinically effective and cost-saving: use of antibiotic-coated catheters compared with use of either antiseptic-coated or standard catheters, maximal sterile barrier precautions during catheter insertion compared with less stringent aseptic technique, and use of chlorhexidine gluconate as either a skin preparation or impregnated into the insertion site dressing compared with use of povidone-iodine skin preparation and nonimpregnated dressings. Results of these evaluations are robust to a wide range of parameter estimates and assumptions. Two other interventions showed health benefits and increased costs: use of a 3-day or 10-day catheter replacement regimen rather than replacement every 5 days and use of commercially available plastic bags for delivery of total parenteral nutrition rather than glass bottles. Conclusions about the cost-effectiveness of these interventions changed with use of different parameters and assumptions.

### Usefulness of Evaluations

We have data on the cost-effectiveness of only 6 interventions. These interventions were evaluated separately and not compared with each other. Furthermore, many other interventions have been shown to be clinically effective but, there are no data on their cost-effectiveness. This finding is not consistent with current guidelines (*2*), which recommend that “it is logical to use multiple strategies concomitantly.” The 100,000 Lives Campaign is also formed on the basis of a group of interventions. The existing economic evidence is therefore incomplete and cannot be used to form a coherent policy for preventing CR-BSI. Infection control practitioners and other decision makers require information on the relative cost-effectiveness of all relevant groups of interventions rather than individual strategies (*8*). A good example of using cost-effectiveness to inform a complete policy is provided by Frazier et al (*33*). They evaluated 21 competing strategies for population-based colorectal cancer screening and included all relevant screening methods and frequencies. This study provides policymakers with complete information in as much as all available choices have been compared.

The failure to specify baseline values (i.e., the value authors believe is most likely) for model parameters is also problematic. Instead of estimating a baseline model and then testing whether the conclusions are robust to high and low values, some authors report all possible results on the basis of all possible values for some parameters. This shifts the responsibility of interpreting the results to the reader. The failure to describe how high and low values were chosen for key parameters (i.e., the double-it and half-it approach) compounds the problem.

### Assessing Quality

There was a lack of transparency in the development of model structure. Model structure may have been driven by availability of data rather than careful review of the natural progression of the disease. This could undermine the external consistency of the evaluations as they appear to users. The choice of short-time horizons and narrow economic perspectives inhibits the usefulness of these evaluations by excluding relevant costs and health outcomes from the analysis. The current evidence may represent a blinkered view of the problem and how it should be managed. This situation in turn reduces the extent to which the value of infection control can be compared with other healthcare spending such as cardiac surgery and diabetes prevention.

The quality of data incorporated in the models is highly variable. The authors of 7 studies (*23*,*24*,*26*–*30*) suggest that their results are compromised by an absence of high-quality or precise information, often for key parameters in the model. This finding leads to some skepticism about the results (*31*). Researchers are attempting to provide better estimates of the health and economic outcomes attributable to CR-BSI (*34*). However, a model should not be criticized on the basis of the quality of data used per se. Rather, it should be judged on the techniques used to identify and incorporate the highest quality appropriate and relevant data possible (*35*) for all parameters, not just those relating to effectiveness. Given the lack of information provided by the authors about this process, a more systematic approach to selecting evidence needs to be introduced. Generic tools such as the hierarchy used here (*19*) are useful to judge evidence quality, but this may need to be supplemented with tools such as the hierarchy of quasi-experimental study designs, given the prevalence of the use of these designs in the infection control literature (*36*). Where multiple pieces of relevant information are available, techniques exist for the synthesis of diverse evidence (*37*).

Given the variations in data quality, selecting the best evidence and then propagating the effect of uncertainty in this evidence to the conclusions drawn are important. A good method is probabilistic sensitivity analysis (*38*). This method was used in 3 evaluations (*23*,*27*,*30*). This technique characterizes parameter estimates as distributions rather than discrete values and conducts multiple simulations of the model that draw different parameter values each time from the distributions. This enables the uncertainty around the costs and benefits of a given intervention to be described and the relative contribution to all uncertainty arising from each parameter to be estimated. The next step, which was not conducted for any evaluation, is to estimate the value of collecting more data to inform these parameters (*39*). This step would be particularly relevant to the key parameters identified in this review. The current methods used to derive estimates of costs and deaths attributable to CR-BSI are subject to some bias and may not make intuitive sense to clinicians (*31*). This issue is problematic because these methods are important components in the model, often driving the changes in costs and benefits, and it is likely this finding partly explains why so many interventions appear cost-saving.

This review has some limitations. Despite use of a broad search strategy, we may not have identified all model-based economic evaluations in this area; some evaluations may not have been published or are available only as abstracts. Also, our assessment of the quality of evaluations using the good practice criteria may reflect the way evaluations are reported rather than conducted. In fact, word limits often prevent authors from providing a full description of methods. However, any indication that a criterion was addressed was taken as an evaluation that met that attribute.

## Conclusion

We do not have a comprehensive understanding of the economics of preventing CR-BSI. Policymakers and regulatory agencies are unable to recommend the best approach to mitigate risks for CR-BSI in patients in intensive-care units. Those who propose to undertake research in this area would benefit from a careful consideration of this review. Modelers should collaborate and aim to develop a consensus on key issues such as model structure, data sources, and evaluation methods. This activity is promoted by the International Society for Pharmacoeconomics and Outcomes Research and The Cancer Intervention and Surveillance Modeling Network. Ultimately, the best policy for preventing CR-BSI will emerge from an iterative process that includes researchers, clinicians, modelers, and decision makers.

## Supplementary Material

Appendix Economic Evaluations Excluded from the Review
