# Supplementary material for: Economic Evaluation and Catheter-related Bloodstream Infections
Source: Emerg Infect Dis. 2007 Jun;13(6):815–23. doi: 10.3201/eid1306.070048 (PMC2792862; doi:10.3201/eid1306.070048)
Supplement: Appendix — Economic Evaluations Excluded from the Review [file 07-0048_app-s1.pdf]

Publisher: CDC; Journal: Emerging Infectious Diseases
Article Type: Perspective; Volume: 13; Issue: 6; Year: 2007; Article ID: 07-0048
DOI: 10.3201/eid1306.070048; TOC Head: Perspective

Appendix. Economic Evaluations Excluded from the Review

Trial-based Economic Evaluations

Borschel DM, Chenoweth CE, Kaufman SR, Hyde KV, van der Elzen KA, Raghunathan TE, et al. Are antiseptic-coated central venous catheters effective in a real-world setting? Am J Infect Control. 2006;34:388–93.

Sutton CD, Garcea G, Pollard C, Berry DP, Dennison AR. The introduction of a nutrition clinical nurse specialist results in a reduction in the rate of catheter sepsis. Clin Nutr. 2005;24:220–3.

Warren DK, Zack JE, Mayfield JL, Chen A, Prentice D, Fraser VJ, et al. The effect of an education program on the incidence of central venous catheter-associated bloodstream infection in a medical ICU. Chest. 2004;126:1612–8.

Coopersmith CM, Rebmann TL, Zack JE, Ward MR, Corcoran RM, Schallom ME, et al. Effect of an education program on decreasing catheter-related bloodstream infections in the surgical intensive care unit. Crit Care Med. 2002;30:59–64.

Slater F. Cost-effective infection control success story: a case presentation. Emerg Infect Dis. 2001;7:293–4.

Sherertz RJ, Ely EW, Westbrook DM, Gledhill KS, Streed SA, Kiger B, et al. Education of physicians-in training can decrease the risk for vascular catheter infection. Ann Intern Med. 2000;132:641–8.

Cowl CT, Weinstock JV, Al-Jurf A, Ephgrave K, Murray JA, Dillon K. Complications and cost associated with parenteral nutrition delivered to hospitalized patients through either subclavian or peripherally-inserted central catheters. Clin Nutr. 2000;19:237–43.

Laura R, Degl'Innocenti M, Mocali M, Alberani F, Boschi S, Giraudi A, et al. Comparison of two different time interval protocols for central venous catheter dressing in bone marrow transplant patients: results of a randomized, multicenter study. The Italian Nurse Bone Marrow Transplant Group (GITMO). Haematologica. 2000;85:275–9.

Meier PA, Fredrickson M, Catney M, Nettleman MD. Impact of a dedicated intravenous therapy team on nosocomial bloodstream infection rates. Am J Infect Control. 1998;26:388–92.

Raad I, Darouiche R, Dupuis J, Abi-Said D, Gabrielli A, Hachem R, et al. Central venous catheters coated with minocycline and rifampin for the prevention of catheter-related colonization and bloodstream infections. A randomized, double-blind trial. The Texas Medical Center Catheter Study Group. Ann Intern Med. 1997;127:267–74.

Maki DG, Stolz SM, Wheeler S, Mermel LA. Prevention of central venous catheter-related bloodstream infection by use of an antiseptic-impregnated catheter. A randomized, controlled trial. Ann Intern Med. 1997;127:257–66.

Howard TJ, Stines CP, O'Connor JA, Schuster WS, Wiebke EA. Cost-effective supply use in permanent central venous catheter operations. Am Surg. 1997;63:441–5.

Long-term Catheters

Melville CA, Bisset WM, Long S, Milla PJ. Counting the cost: hospital versus home: central venous catheter survival. J Hosp Infect. 1997;35:197–205.

Mokrzycki MH, Singhal A. Cost-effectiveness of three strategies of managing tunnelled, cuffed haemodialysis catheters in clinically mild or asymptomatic bacteraemias. Nephrol Dial Transplant. 2002;17:2196–203.

Johnson DW, MacGinley R, Kay TD, Hawley CM, Campbell SB, Isbel NM, et al. A randomized controlled trial of topical exit site mupirocin application in patients with tunnelled, cuffed haemodialysis catheters. Nephrol Dial Transplant. 2002;17:1802–7.

Focus not on CR-BSI Prevention

Siegman-Igra Y, Anglim AM, Shapiro DE, Adal KA, Strain BA, Farr BM. Diagnosis of vascular catheter-related bloodstream infection: a meta-analysis. J Clin Microbiol. 1997;35:928–36.

Cox CE, Carson SS, Biddle AK. Cost-effectiveness of ultrasound in preventing femoral venous catheter-associated pulmonary embolism. Am J Respir Crit Care Med. 2003;168:1481–7.

Rosen AB, Fowler VG Jr, Corey GR, Downs SM, Biddle AK, Li J, et al. Cost-effectiveness of transesophageal echocardiography to determine the duration of therapy for intravascular catheter-associated Staphylococcus aureus bacteremia. Ann Intern Med. 1999;130:810–20.

Letter Only

Frank U, Chojnacki T, Dettenkofer M, Daschner FD. Cost-effectiveness of an antiseptic-impregnated central venous catheter in the ICU. Intensive Care Med. 2003;29:139.
